# TET-mediated 5hmC in breast cancer: mechanism and clinical potential

**DOI:** 10.1080/15592294.2025.2473250

**Published:** 2025-02-27

**Authors:** Jiahang Zhang, Nadire Aishan, Zhongqiu Zheng, Siwei Ju, Qina He, Qingna Meng, Xixi Lin, Jiaheng Lang, Jichun Zhou, Yongxia Chen, Bojian Xie, Yangjun Cai, Feiyang Ji, Linbo Wang

**Affiliations:** a Department of Surgical Oncology, Sir Run Run Shaw Hospital, Zhejiang University School of Medicine, Hangzhou, Zhejiang, China; b Provincial Clinical Research Center for CANCER, Hangzhou, Zhejiang, China; c Department of Breast and Thyroid Surgery, Taizhou Hospital of Zhejiang Province, Taizhou, Zhejiang, China

**Keywords:** Breast cancer, 5hmC, TET protein

## Abstract

Breast cancer is the most common cancer among women, with differences in clinical features due to its distinct molecular subtypes. Current studies have demonstrated that epigenetic modifications play a crucial role in regulating the progression of breast cancer. Among these mechanisms, DNA demethylation and its reverse process have been studied extensively for their roles in activating or silencing cancer related gene expression. Specifically, Ten-Eleven Translocation (TET) enzymes are involved in the conversion process from 5-methylcytosine (5mC) to 5-hydroxymethylcytosine (5hmC), which results in a significant difference in the global level of 5hmC in breast cancer compared with normal tissues. In this review, we summarize the functions of TET proteins and the regulated 5hmC levels in the pathogenesis of breast cancer. Discussions on the clinical values of 5hmC in early diagnosis and the prediction of prognosis are also mentioned.

## Introduction

Breast cancer has already surpassed lung cancer as the most prevalent malignancy in women since 2020, and it is one of the leading causes of cancer-related death around the world [1,2]. According to the different expression levels of estrogen receptor (ER), progesterone receptor (PR), human epidermal growth factor receptor 2 (HER2) and Ki67, breast cancer could be generally classified into four molecular subtypes [3]. Pivotal therapeutic advances, such as the use of tamoxifen and trastuzumab, have significantly prolonged survival for patients with ER-positive or HER2-positive tumors [3]. However, many patients fail to respond to these treatments due to tumor heterogeneity. Thus, novel molecular targets involved in tumor progression should be further explored from genetic and epigenetic perspectives.

Epigenetic mechanisms play a significant role in regulating cancer biology. In breast cancer, genes with abnormal methylation status that are involved in essential signal pathways such as WNT and Hedgehog could lead to cancer metastasis and endocrine drug resistance [4]. In addition, the discovery of DNA demethylation and novel oxidative derivations of cytosine, including 5-hydroxymethylcytosine (5hmC), 5-formylcytosine (5fC), and 5-carboxylcytosine (5caC), may offer new strategies for cancer research [5] However, as a key intermediate product of demethylation process, the role of 5hmC in cancer progression is still under investigation. Here we present the mechanisms of TET-mediated DNA hydroxymethylation in breast cancer and discuss their potential values in clinical applications.

## 5hmC and TETs family

DNA cytosine methylation has been revealed to exert multiple influences on various physiological and pathological processes [6]. However, this relatively stable epigenetic modification can be reversed to its unmodified form by either active or passive pathways. In both processes, 5mC is initially oxidized by TET proteins into 5hmC. And it continues via a replication-dependent passive pathway or through thymine DNA glycosylase (TDG) and base excision repair (BER) active pathway [5].

As a relatively robust intermediate product, 5hmC presents specificity in both tissue and genomic distribution. 5hmC is strongly enriched in brain and bone marrow in mammals compared with other adult organs. To be specific, 5hmC is about 40% as much as 5mC in mouse Purkinje neurons, whereas in liver or lung, the level of 5hmC is less than half of that in the central nervous system (CNS) [7,8]. Meanwhile it appears massively in embryonic stem cells (ESCs) and reoccurs during the generation of induced pluripotent stem cells but decreases during differentiation, which represents as a prominent epigenetic feature of multi-lineage potency [9,10]. Genome-wide sequencing suggests that in ESCs, 5hmC is mainly concentrated in exons and near transcriptional start sites (TSSs), which indicates its potential function in transcriptional regulation [9]. In human brain, the content of 5hmC is higher in promoters than in the gene bodies [11]. Scientists also achieved genome-wide profiles of 5hmC in human normal breast tissues and discovered that 5hmC was gathered among breast-specific enhancers and transcriptionally active regions, which were closely correlated to lactate oxidation and immune-related signaling pathways [12].

TET (Ten-Eleven-Translocation) family proteins are iron(II)/α-ketoglutarate-dependent dioxygenases, which oxidize 5mC to three demethylated derivatives including 5hmC, 5fC and 5caC [13]. Among these, 5hmC is the most stable oxidation form, as the other derivatives are excised from the DNA by base excision repair enzymes [13]. Among the three currently known TET proteins, full-length TET1 and TET3 rather than TET2 contain a CXXC domain at the amino terminus and their core catalytic domains at the carboxyl terminus are composed of a double-stranded β-helix (DSBH) domain and a cysteine-rich domain [14]. Recently, Scientists identified a novel TET1 isoform, TET1^ALT^ [15]. This isoform lacks the CXXC DNA binding domain and exhibits an anemic effect on DNA methylation compared to full-length TET1. TET1^ALT^ is aberrantly expressed in various cancers, and its activation is associated with poor prognosis in breast cancer, glioblastoma, and uterine cancer [15].

Since the role of methylation has been studied extensively as an epigenetic modification involved in gene regulation and tumorigenesis, scientists wonder whether its reverse process, hydroxymethylation, has synergistic or opposing effects during cancer development. Massive researches underpinned the view that 5hmC levels are significantly reduced in various cancers compared to normal tissues [16–18]. Interestingly, no significant correlation was found between the levels of 5hmC and 5mC in cancer tissues, suggesting that the decrease in 5hmC may result from a variety of signaling pathways rather than being directly linked to 5mC levels alone [16]. For instance, scientists found that 5hmC levels is associated with TP53 mutation in pancreatic ductal adenocarcinoma [19]. Additionally, aberrant metabolism in cancer cells could also influence the function of TET proteins, leading to global changes of 5hmC [20]. In conclusion, TET-regulated hydroxymethylation is involved in several key tumorigenesis-related signaling pathways. It may offer new strategies for cancer diagnosis, prognosis and even drug resistance issues.

## Detection methods of 5hmC

Since genome-wide hydroxymethylation modifications are less abundant than methylation modifications, distinguishing 5hmC from 5mC and unmodified cytosine is crucial for enhancing the specificity and accuracy of 5hmC detection [11]. Scientists have developed a variety of multidisciplinary approaches using chemistry, biology and physics to detect 5hmC from the genome-wide level or single base level. Current widely used methods can be generally summarized into five categories: 1. methods based on antibodies; 2. liquid chromatography with mass spectrometry (LC-MS); 3. chemical methods based on bisulfite conversion; 4. enzyme-assisted methods; 5. innovative methods.

Traditional antibody-based methods, such as 5hmC DNA immunoprecipitation (DIP) and ELISA, have the advantages of simple operation, low DNA requirement and high sensitivity [21]. However, 5hmC-DIP fails to provide single-base resolution of DNA modification sites and there is a risk of non-specific binding to DNA. Chromatographic methods can achieve the most sensitive and accurate detection of global 5hmC level [5]. But such methods are expensive and require specialized knowledge for sample operation and data analysis.

In order to reduce costs and simplify detection steps, scientists developed a series of chemical transformation methods including bisulfite conversion, oxidation of 5hmC and 5hmC glycosylation [22,23]. CMS method converts 5hmC to cytosine 5-methylenesulfonate, effectively distinguishing 5hmC from 5mC [9]. GLIB method uses biotinylated glucose labeling and affinity-based enrichment, which increases the specificity for 5hmC detection and simplifies the experimental process [22]. Scientists subsequently explored new methods to analyze locus-specific 5hmC. Oxidative bisulfite sequencing (oxBS-Seq) method can oxidize 5hmC to 5fC, followed by bisulfite conversion of 5fC and cytosine to uracil [23]. By comparing the results of bisulfite sequencing (BS-Seq), oxBS-Seq can accurately detect 5hmC sites. In addition, Tet-assisted bisulfite sequencing (TAB-Seq) also achieves single-base resolution detection of 5hmC by combining TET enzyme oxidation and bisulfite treatment [24]. However, bisulfite methods often cause DNA degradation and introduce side chemical reactions, limiting its application in rare samples or large studies.

To solve these problems, bisulfite-free approaches such as APOBEC-coupled epigenetic sequencing (ACE-seq), 5hmC-CATCH and enzymatic methyl-seq (EM-seq) were developed, which reduced the DNA input and significantly enhanced the detection efficiency [25–27]. 5hmC-Seal was also invented to capture and label 5hmC in very small amounts of DNA, such as circulating cell-free DNA (cfDNA) [28]. The TET-assisted pyridine borane sequencing (TAPS) and its derived methods TAPSβ and CAPS could achieve the detection of unmodified cytosine, 5mC and 5hmC respectively [29,30]. Further innovative methods used SSD-Seq or Bt-AuNP-G to detect 5hmC modifications directly, but they were not widely promoted because of the expensive equipment and engineered materials [31,32]. Recently the Joint-snhmC-seq method realized the profiling of both 5hmC and 5mC in single cells, which uncovered the epigenetic heterogeneity among different cells [33]. Here we summarized the advantages and disadvantages of approaches for detecting the hydroxymethylation modifications, as shown in Table 1.

**Table 1.** Approaches for detecting 5hmC.

References example	Method	Material	Transformation process	Advantages	Disadvantages		Sample	
Jin et al. [11]	5hmC-DIP	anti-5hmC antibody		high sensitivity, low input, simple operation	not single-base level, depend on antibody quality	human brain		
Ito et al. [5]	HPLC-MS/MS	electrospray ionization mode		high accuracy and sensitivity,	expensive, specialized knowledge requirement	mouse ESC, mouse organs		293 cells
Song et al. [22]	glycosylation & chemical labeling	β-GT, UDP-6-N3-Glu, cyclooctyne-biotin	5hmC/biotin-N3–5-gmc	high specificity, low input, easy operation	limited sensitivity, depend on labeling and binding efficiency	mouse ESC, mouse aNSC		HeLa cells, HEK293FT cells
Pastor et al. [9]	GLIB	β-GT, UDP-glucose, sodium periodate, ARP	5hmC/biotin-5hmC	high specificity, low input, easy operation	limited sensitivity, low resolution,	mouse ESC		
	BS-CMS	sodium bisulphite	5hmC/CMS	high sensitivity and accuracy	complex operation, expensive			
Booth et al. [23]	BS-Seq	bisulfite	5hmC/CMS/C5mC/CC/U/T	single-base resolution, BS – oxBS = true 5hmC	DNA degradation, complex operation	mouse ESC		
	oxBS-Seq	KRuO4 bisulfite	5hmC/5fC/U/T5mC/CC/U/T					
Yu et al. [24]	TAB-Seq	β-GT, mouse TET1, bisulfite	5hmC/5ghmC/C5mC/5caC/5caU/T	single-base resolution	complex operation,expensive	mouse ESC		human ESC
Li et al. [28]	5hmC-Seal	β-GT, UDP-6-N3-Glu,	5hmC/biotin-N3–5-gmc	minimal DNA input, apply on cfDNA	not single-base resolution, depend on labeling and binding efficiency			plasma from patients with colorectal, gastric, pancreatic, liver or thyroid cancer and normal tissue
Schutsky et al. [25]	ACE-seq	β-GT, A3A	5hmC/5ghmC/C5mC/U/TC/U/T	bisulfite-free method, high sensitivity, low input	limited specificity,depend on enzyme	murine cortical, mouse ESC, mouse excitatory neuron (*NeuroD6/NEX+*)		
Zeng et al. [26]	hmC-CATCH	KRuO4,Biotin,5fC blockage	5hmC/5fC	bisulfite-free method, nanoscale input,	depend on labeling, limited protocols			human ESC, cfDNA
Vaisvila et al. [27]	EM-Seq	TET2, β-GT, A3A	5hmC/5ghmC 5mC/5caC C/U	purely enzymatic method, high sensitivity, low input	need to combine other methods to detect 5hmC, depend on enzyme	mouse ESC		cfDNA, lung FFPE DNA
Liu et al. [29,30]	TAPS, TAPSβ, CAPS	PB, β-GT, KRuO4, TET	5hmC/5fC/DHU 5mC/5caC/DHU	distinguish C, 5mC and 5hmC separately	depend on enzyme, complex operation	mouse ESC		
Xie et al. [31]	SSD-seq	eA3A-v10	5hmC/C 5mC/T C/U/T	single-step process, bisulfite or glycosylation free	expensive,			human lung tissue
Imran et al. [32]	Bt-AuNP-G method	Bt-AuNPs, Graphene sheet	5hmC/5fC	one-step electrochemical process, highly sensitive, label-free detection	expensive	murine tissues including brain, liver, heart, spleen and thymus, mouse models with HCC		prostate cancer cell (C4-2B), normal prostate epithelial cell (RWPE-1)
Fabyanic et al. [33]	Joint-snhmC-seq	bisulfite A3A	5hmC/CMS 5mC/T C/U	single cell level	expensive	mouse cortex		

β-GT β-glucosyltransferase, ESC Embryonic stem cell, aNSC Adult neural stem cell, ARP Aldehyde reactive probe, CMS Cytosine 5-methylenesulphonate, cfDNA Cell-free DNA, A3A AID/APOBEC3A, FFPE Formalin-fixed paraffin-embedded, PB Pyridine borane, DHU Dihydrouracil, Bt-AuNPs Biotin and gold nanoparticles, HCC Hepatocellular carcinoma.

## Current status of 5-hmC research in breast cancer

### Milestones of the research for 5hmC/TET in breast cancer

**Figure 1.**
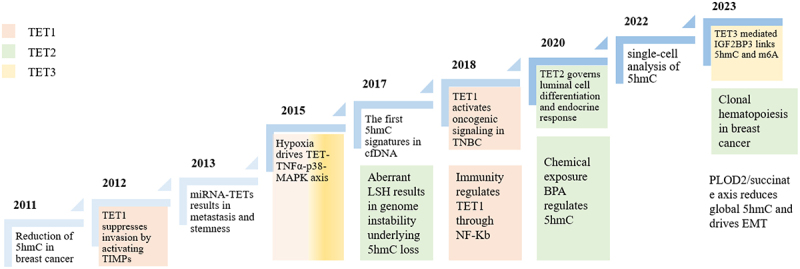
Major advances of 5hmC and different TET family proteins in breast cancer.

Several studies have indicated that 5hmC and TET enzymes have a significant impact on the occurrence and development of breast cancer [34]. In 2011, Haffner et al. first elucidated the profound decrease of 5hmC in breast cancer than in the adjacent normal tissues [35]. In 2012, Hsu and his team discovered that TET1 suppressed tumour invasion and metastasis in breast cancer by deterring the genes of tissue inhibitors of metalloproteinases from being methylated [36]. Later, articles reported that the miR-22-TETs-miR-200 axis played an essential role in EMT, invasion and metastasis of breast cancer [37]. In 2015, Wu et al. discovered that under environmental stress, particularly hypoxia, TET1 and TET3 were deregulated, leading to a genome-wide reduction in DNA hydroxymethylation in breast cancer [38]. Subsequently, the first circulating cell-free 5hmC signatures emerged, highlighting the potential of epigenetics in tumour diagnosis [39]. However, breast cancer did not show distinctive signatures compared to the normal tissue. In 2008, research revealed that immunity played a role in TET1 regulation and influenced cancer cell epigenetics [40]. Additionally, scientists identified an interaction between TET2 and estrogen receptor α (ERα), offering a potential explanation for issues related to endocrine resistance [41,42]. With the advent of single-cell sequencing era, researchers developed strategies to measure 5hmC/5hmU levels in individual cells and thus uncovered the heterogeneous signatures among distinctive cancer cell types [43,44]. Herein, we summarize the major advances in understanding the roles of TET proteins and 5hmC in breast cancer (Figure 1).

### Potential in hormonal treatment resistance

According to the latest studies, hormone receptor-positive breast cancer still accounts for the highest proportion of all molecular types, thus giving priority to endocrine therapy [45]. As a ligand-dependent nucleic binding protein, ER not only regulates the transcription of its downstream estrogen-responsive genes by binding directly to a specific DNA sequence called estrogen response element (ERE), but also functions as a non-canonical RNA-binding protein, which sustains the tamoxifen resistance [46,47]. Researchers have already discovered several particular modes of regulation and interaction between ER and TET2, which may provide novel strategies to endocrine resistance [42,48].

An original feedback loop between ERα and TET2 has been identified. In MCF7 cell lines, the activated ERα could firstly promote the expression of DNA methyltransferase (DNMT), which further mediated the promoter methylation of TET2, resulting in gene silencing as well as low levels of DNA hydroxymethylation in breast cancer [49]. ER could also promote TET2 expression by combining to its enhancers and this activity could be interrupted by tamoxifen treatment, which perhaps raised a conjecture to explain the endocrine resistance [41]. Meanwhile, TET2 could also regulate ER expression. Both in vivo and in vitro tests show that TET2 and FOXP1 complex promotes the demethylation of *ESR1, GATA3*, and *FOXA1*, which are usually silenced in advanced breast cancer [42]. Moreover, in TET2-knockout breast cancer mice models, ER expression decreased considerably, which was in line with the endocrine resistance condition [42].

**Figure 2.**
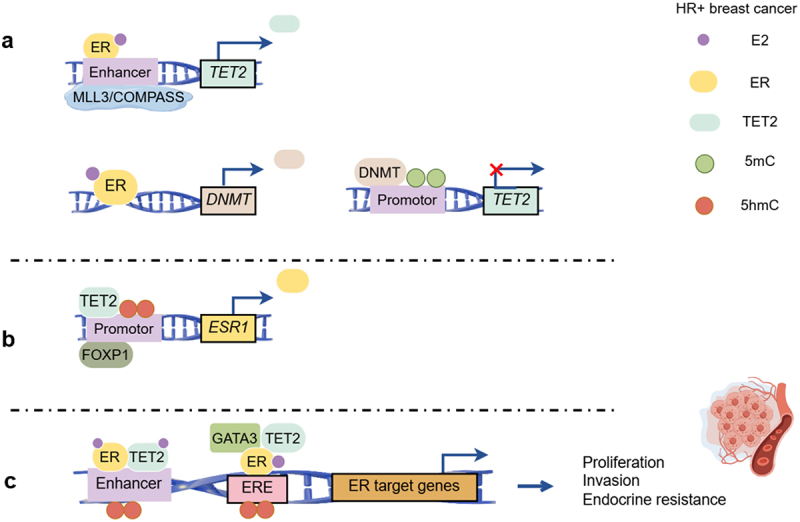
ER and TET2 regulate each other in gene transcription levels in hormone receptor-positive (HR+) breast cancer. a). ER could either promote or inhibit TET2 expression after activated by E2. b). TET2 regulates ER expression through the demethylation of *ESR1* promoter. c). As the target of E2, ER and TET2 work synergistically to activate er-related downstream signaling pathways, which are involved in tumor proliferation and invasion.

As a direct target of estradiol (E2), TET2 worked synergistically on downstream estrogen signaling pathways by recruiting ERα to active enhancers. Besides, TET2 could be recruited by the key ER-associated transcription factor GATA3 and acted as a component of the ER complex [50]. However, ER was inactivated due to E2 deprivation, followed by TET2 reduction, as well as the DNA hypermethylation and histone deacetylation in enhancers [48]. Thus, ER-related cofactors, such as AP-1 and FOX, failed to bind to their target regions, which may probably cause endocrine resistance [48]. Loss of TET2 caused an impressive decrease in the 5hmC levels, especially at ER cis-regulatory elements, which emphasized again the essential role of TET2 in mediating the transcription of ER target genes particularly in breast cancer cells [50]. Here we depict the ER-TET2 feedback loop exclusively in hormone receptor-positive breast cancer (Figure 2).

### Regulations in tumor microenvironment

The role of epigenetic regulation in tumor immune microenvironment (TIME) is one of the current research priorities in cancer. Different TET family proteins could act conversely in terms of immune responses in different types of breast cancer. Studies have shown that TET2 mediates DNA demethylation of the immune checkpoint HLA-G in MCF7 cells, leading to its overexpression [51]. In basal-like breast cancer (BLBC), overall TET1 expression is elevated compared to normal tissue, and is negatively correlated with levels of immune defense markers and cells. In vitro studies further confirmed that activated NF-κB, a key immunoregulatory transcription factor, directly regulates TET1 by binding to its promoter [40]. This novel immune-driven regulation of TET1 builds a link between immunity and cancer epigenetics, providing insights into epigenetic drugs targeting the immune system.

Studies concerning cancer metabolism variations began to prevail since Warburg first proposed the theory of aerobic glycolysis [52]. Metabolic pathways such as tumor oxidative metabolism and lipid metabolism have attracted much attention. As one of the major characteristics of TME, hypoxia is considered as a key factor in cancer progression, influencing the process of apoptosis, autophagy, DNA damage and drug efflux [53]. There is also a link between hypoxia and epigenetic changes. Under hypoxic conditions, 5hmC decreased at gene promoters and enhancers in both MCF7 and MCF10A cell lines, due to a decreased TET hydroxylase function [54]. Similar results were also shown in mice and human tumor tissues, and it suggested that hypoxia could lead to hypermethylation events, especially in tumor suppressor gene promoters [54]. Hypoxia-inducible transcription factors (HIFs) mediated cellular responses to hypoxia in cancer by igniting several downstream signaling pathways, such as VEDF, estrogen and Notch [55]. Both in vivo and in vitro studies showed that DNA demethylation could facilitate HIFs binding activity, leading to high immune checkpoint expression and tumor immunotolerance [56]. Researchers also discovered that overexpressed HIF1α upregulates TET1/TET3 through binding to their promoters, resulting in a global increase of 5hmC. This increase is associated with the activation of TNFα-p38-MAPK signaling axis, which finally causes breast tumor-initiating cell (BTIC) properties [36]. Another study reported that TET3 activity is impaired by hypoxia, resulting in the loss of hydroxymethylation at the E2F1 transcription factor binding region. This loss contributes to decreased levels of epithelial splicing regulatory protein 1 (ESRP1), an RNA-binding protein critical for the epithelial-mesenchymal transition (EMT) in cancer [57].

In terms of lipid metabolism, obesity has been reported as a risk factor for breast cancer, especially in the triple-negative subtype [58]. Studies found that TET1 upregulated TAR DNA-binding protein (TARDBP) and enhanced the function of obesity-driven cancer stem cells in triple-negative breast cancer (TNBC) [59]. Additionally, the aberrant accumulation of succinate, a potential new hallmark of cancer, is associated with EMT, cancer stemness, and the decrease of 5hmC level. Researchers demonstrated that silencing *PLOD2* gene in MCF7 cells decreased cytoplasmic succinate levels but elevated 5hmC levels instead, which may at least partially prevent cancer progression [60]. Further novel therapeutic strategies targeting tumor metabolism are being explored. For instance, the α-ketoglutarate dehydrogenase (α-KGDH) inhibitor, also called AA6, not only increase the activity of TETs, but also intrigue the TET-miR200-Zeb1/CtBP1-MMP3 axis, which impedes EMT and breast cancer-associated lung metastasis [61].

### Improved stemness in cancer

Scientists have discovered that terminally differentiated cells exhibit high levels of 5hmC, while stem cells and progenitor cells showed reduced 5hmC levels, a pattern similarly observed in breast cancer cells [35]. Therefore, researchers speculated that the stem-like characteristics of tumor cells may be linked to their hydroxymethylation levels. Different TET family members have different effects on cancer stemness. For instance, TET1 and TET3 play a positive role in maintaining breast tumor-initiating cell phenotypes through the TET-TNFα-p38-MAPK axis, as evidenced by the CD44^+^/CD24^−^ expression, a recognized biomarker of breast cancer stem cells [38]. However, in vitro studies showed that TET2-activated miR200c downregulates PKCζ, contributing to a decrease of cancer stem cell pool [62]. Further in vitro and in vivo studies revealed that TET2 could be silenced by miR22, resulting in hypermethylation of the miR200c promoter and enhanced cancer stemness [37]. Another study also suggested that mouse models with TET2 deletion demonstrated an increased self-renewal capacity of mammary stem cells (MaSCs) [42].

### Crucial genes in tumor proliferation, invasion and metastasis

The diverse functions of TET proteins are reflected in their involvement in the epigenetic regulation of crucial tumor suppressor genes and various cancer-related signaling pathways, resulting in tumorigenesis and progression. In tumor tissues, researchers found that reduced TET1 expression led to lower levels of 5hmC and mRNA for *LZTS1*, a well-known tumor suppressor gene associated with breast cancer progression and metastasis [63]. In TNBC cell lines, the transcription of TET2 could also be repressed by the p65-KDM2A complex, which hampered the expression of E-cadherin and EpCAM, thereby promoting cancer migration [64]. Additionally, researchers discovered a profound relationship between 5hmC and N6-methyladenosine (m6A), a reversible modification of mRNAs. TET3 could upregulate IGF2BP3, an essential reader of m6A, leading to mRNA instability of a tumor suppressor gene NF1 and tumor progression in TNBC [65].

Interestingly, while most studies reported low TET expression in tumors, positioning TET proteins as tumor suppressors, one study identified TET1 as a potential oncogene [66–68]. TET1 was shown to promote cancer proliferation by mediating hypomethylation of genes involved in oncogenic pathways such as PI3K, EGFR, and PDGF. Using CRISPR-Cas9 to knock out TET1 in TNBC cell lines resulted in increased immune responses, downregulation of PI3K pathway genes, and significantly reduced cell proliferation [68].

Epithelial-mesenchymal transition (EMT) is a critical initial step in the process of tumor invasion and metastasis [69]. Proteins such as matrix metalloproteinases (MMPs) play a significant role in the interactions between cancer cells and the extracellular matrix [70]. Hsu et al. reported that TET1 inhibits cancer invasion by regulating the tissue inhibitors of metalloproteinases (TIMPs) through DNA demethylation [36]. In addition, TET1 depletion could impair the favorable effect of TSA (an inhibitor of histone deacetylases) in suppressing breast cancer invasion [71]. Many studies have indicated that the genomic landscape of breast cancer alters during progression and metastasis [72]. Wu et al. found that metastatic lymph node lesions generally display lower 5hmC levels than primary tumor foci in patient samples [73]. This difference may be concerned with cancer cell adaptation in new microenvironments and further dissemination to distant organs. They also identified MAP7D1, regulated by TET1, as a novel biomarker for predicting lymph node metastasis in breast cancer [73]. Sun et al. discovered the HMGA2-TET1-HOXA9 axis, which regulates tumor proliferation and metastasis, further highlighting its potential as a prognostic signature [74]. Moreover, metastatic tissues often show greater downregulation of TET2 and 5hmC levels compared to non-metastatic tissues. This can be explained by the aberrant lymphoid specific helicase (LSH), a chromatin remodeling factor which alters 5hmC levels in pericentromeric satellite repeats, contributing to microsatellite stability [75]. These findings underline the significant value of 5hmC as a biomarker for predicting metastasis and provide insights into mechanisms of genome instability driven by epigenetic changes.

## The value of 5hmC and TETs in clinical applications

### Environmental pathogenic evidence

Breast cancer pathogenesis is highly diverse, influenced by both internal biological factors and external environmental elements. Several studies have highlighted that harmful environmental organic compounds can regulate demethylation via TET proteins, contributing to tumorigenesis [49,76]. For instance, Li and colleagues demonstrated that exposure to bisphenol A (BPA), a ubiquitous environmental chemical, affects DNA hydroxymethylation and promotes cell proliferation by targeting estrogen receptors in breast cancer [49]. Additionally, animal experiments revealed that the herbicide glyphosate induces tumor development by triggering TET3-mediated DNA demethylation and it could leave long-lasting effects even after a removal of glyphosate. Interestingly, after performing primary cell culture on the lumps obtained from these animals, they identified an ER positive phenotype, which was sensitive to tamoxifen and exhibited invasive and migratory potentials [76]. Although these in vitro studies provide subtle clues for the relationship between environmental exposures and DNA hydroxymethylation in breast cancer, further epidemiological evidence is needed.

### Early diagnosis of breast cancer

Recent researches suggest that both TET proteins and 5hmC levels hold potential to predict the onset of breast cancer [66,67]. It is widely recognized that ductal carcinoma in situ (DCIS) and breast ductal intraepithelial neoplasia (DIN) are precancerous lesions. Consequently, some researchers have investigated the utility of 5hmC in identifying early pathological changes. Zhang et al. compared the three groups: DIN, DCIS with microinvasion (DCIS-MI) and invasive breast cancer, finding that 5hmC and TET2 levels were inversely correlated with histological grade. This highlights the potential of 5hmC as a reliable biomarker for early diagnosis [67]. Other studies found that even small lesions with low histological grades displayed a huge reduction in 5hmC, suggesting its value as an early diagnostic marker [35]. However, a recent study utilizing a solid-state nanopore assay, capable of directly analyzing global 5hmC levels, found no significant difference between normal tissue and stage 1 breast cancer tissue [77]. These findings suggest that changes in 5hmC levels may occur as a downstream consequence of tumorigenesis rather than as an early event. Nevertheless, because the novel technique used in this study has not yet been widely adopted, it is premature to dismiss the potential value of 5hmC in early cancer screening. Further research and evidence are needed to clarify its role.

Detection of trace amounts of cell-free DNA (cfDNA) and other tumor biomarkers in circulating blood plasma has emerged as a promising method for early cancers screening. The advent of next-generation sequencing (NGS) technologies has enabled more precise and comprehensive profiling of tumor genetic and epigenetic information. Studies have shown that analyzing 5hmC in cfDNA can successfully detect early-stage cancers, such as hepatocellular carcinoma and pancreatic cancer [78,79]. Also, 5hmC-based biomarkers were reported to hold equal authenticity as conventional tissue biomarkers in gastric cancer [28]. However, the application of 5hmC in breast cancer still needs improvement. Song et al. conducted 5hmC sequencing in cfDNA from pan-cancers, suggesting that these signatures are correlate with tumor stages, especially in lung cancer. But their *t*SNE analysis failed to separate breast cancer samples from healthy controls, which may due to a limited sample size [39].

### Prediction of disease prognosis

Several studies indicated that the decreased TET expression and reduced 5hmC levels were associated with poor patient outcomes [18,66,80]. Yang et al. reported that breast cancer patients with high TET1 mRNA levels exhibited better overall survival (OS) [66]. However, the result drawn using Kaplan-Meier survival analysis is not reliable, because it cannot rule out the influence of other interfering factors, such as tumor size, stage and molecular subtypes. Besides, the relationship between TET protein levels and prognosis should be further evaluated to supplement this conclusion. Interestingly, Mitrea et al. integrated 5hmC data and concluded that low 5hmC levels, consistent with reduced TET1 mRNA expression, were linked to better outcomes in TNBC patients – a conclusion entirely opposite to previous reports [81]. This result reflects that TET family members play distinctive roles in different molecular subtypes of breast cancer.

Furthermore, TET proteins and 5hmC levels have been implicated in predicting chemotherapy efficacy. Higher TET3 and TDG mRNA levels correlated with improved OS in patients undergoing anthracycline therapy after mammary surgery, establishing these markers as independent prognostic factors [66]. In addition, both 5hmC and 5mC play a role in predicting breast cancer prognosis. Decreased 5hmC was associated with poor disease-free survival (DFS) in ER/PR-negative subtypes, while reduced 5hmC was observed in hormone receptor-positive subtypes [18].

## Conclusion and future perspectives

**Figure 3.**
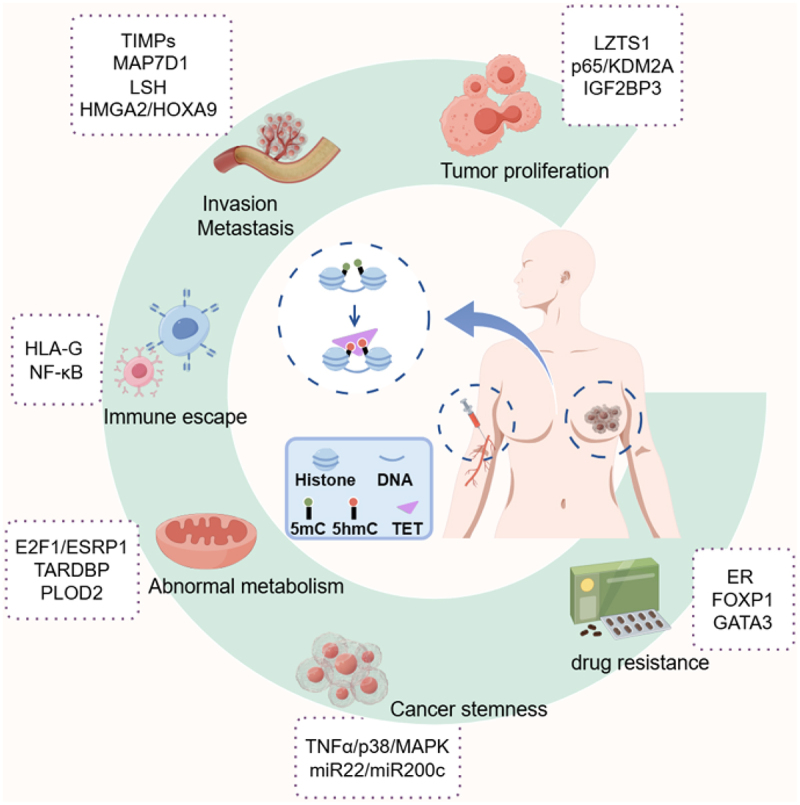
5hmC can be detected from tissues and blood samples of breast cancer patients. The aberrant hydroxymethylation of essential genes and dysregulated TET proteins play a crucial role in regulating tumor proliferation, immune response, abnormal metabolism, cancer stemness and drug resistance.

DNA hydroxymethylation is a burgeoning area of epigenetic research, which plays an essential role in genome programming. Accumulating evidence highlights the extensive involvement of 5hmC in tumorigenesis and progression, offering valuable insights for clinical diagnosis and prognosis prediction. In breast cancer, 5hmC modification mediated by TET proteins influence tumor invasion and metastasis through their role in regulating tumor immunity, metabolism, cancer stemness, and drug resistance (Figure 3).

Interestingly, the role of the TET family as oncogenes or tumor suppressor genes remains ambiguous, as the functions of TET1, TET2, and TET3 vary significantly across different molecular subtypes of breast cancer. Beyond their involvement in oncogenesis and tumor progression, 5hmC levels and TET protein expression are being explored for their potential in early cancer diagnosis and in assessing the risk of distant recurrence and metastasis. Detecting 5hmC in cfDNA also represents a promising biomarker technique in clinical application. However, this approach has limitations in breast cancer, as no significant differences in 5hmC levels have been observed between patients and healthy individuals, indicating the need for multi-omics integration to enhance diagnostic accuracy in the future. Moreover, the development of single-cell nucleic acid modification detection technologies addresses the challenge of tumor heterogeneity and provides precise therapeutic targets. In summary, 5hmC and TETs hold potential as novel epigenetic biomarkers for tumor diagnosis and prognosis. However, further studies are still needed to clarify their distinct roles across different molecular subtypes of breast cancer.

## Abbreviation

5hmC: 5-hydroxymethylcytosine
5mC: 5-methylcytosine
A3A: AID/APOBEC3A
AMPK: AMP-activated kinase
aNSC: Adult neural stem cell
ARP: Aldehyde reactive probe
BER: Base excision repair
BLBC: Basal-like breast cancer
BTIC: Breast tumor-initiating cell
Bt-AuNPs: Biotin and gold nanoparticles
cfDNA: Cell-free DNA
CMS: Cytosine 5-methylenesulphonate
CNS: Central nervous system
DHU: Dihydrouracil
DNMT: DNA methyltransferase
DSBH: Double-stranded β-helix
ESC: Embryonic stem cell
EMT: Epithelial mesenchymal transition
ERE: Estrogen response element
ER: Estrogen receptor
ESC: Embryonic stem cell
FFPE: Formalin-fixed paraffin-embedded
HCC: Hepatocellular carcinoma
HER2: Human epidermal growth factor receptor2
HIF: Hypoxia-inducible transcription factor
HR+BC: Hormone receptor-positive breast cancer
IDH1: Isocitrate dehydrogenase-1
PB: Pyridine borane
PR: Progesterone receptor
TDG: Thymine DNA glycosylase
TET: Ten-Eleven-Translocation
TIMP: Tissue inhibitors of metalloproteinase
TME: Tumor microenvironment
TNBC: Triple-negative breast cancer
TSS: Transcriptional start site
α-KG: α-ketoglutarate
β-GT: β-glucosyltransferase
